# *In situ* Phenotyping of Grapevine Root System Architecture by 2D or 3D Imaging: Advantages and Limits of Three Cultivation Methods

**DOI:** 10.3389/fpls.2021.638688

**Published:** 2021-06-29

**Authors:** Yuko Krzyzaniak, Frédéric Cointault, Camille Loupiac, Eric Bernaud, Frédéric Ott, Christophe Salon, Anthony Laybros, Simeng Han, Marie-Claire Héloir, Marielle Adrian, Sophie Trouvelot

**Affiliations:** ^1^Agroécologie, AgroSup Dijon, CNRS, INRAE, Université de Bourgogne, Université de Bourgogne Franche-Comté, Dijon, France; ^2^UMR A 02-102 PAM Université de Bourgogne-Franche Comté, AgroSup Dijon, Dijon, France; ^3^Laboratoire Léon Brillouin, UMR 12 CEA-CNRS, CEA Saclay, Gif-sur-Yvette, France

**Keywords:** root system architecture, root traits, grapevine, phenotyping, rhizotron, neutron tomography, 2D/3D imaging

## Abstract

The root system plays an essential role in the development and physiology of the plant, as well as in its response to various stresses. However, it is often insufficiently studied, mainly because it is difficult to visualize. For grapevine, a plant of major economic interest, there is a growing need to study the root system, in particular to assess its resistance to biotic and abiotic stresses, understand the decline that may affect it, and identify new ecofriendly production systems. In this context, we have evaluated and compared three distinct growing methods (hydroponics, plane, and cylindric rhizotrons) in order to describe relevant architectural root traits of grapevine cuttings (mode of grapevine propagation), and also two 2D- (hydroponics and rhizotron) and one 3D- (neutron tomography) imaging techniques for visualization and quantification of roots. We observed that hydroponics tubes are a system easy to implement but do not allow the direct quantification of root traits over time, conversely to 2D imaging in rhizotron. We demonstrated that neutron tomography is relevant to quantify the root volume. We have also produced a new automated analysis method of digital photographs, adapted for identifying adventitious roots as a feature of root architecture in rhizotrons. This method integrates image segmentation, skeletonization, detection of adventitious root skeleton, and adventitious root reconstruction. Although this study was targeted to grapevine, most of the results obtained could be extended to other plants propagated by cuttings. Image analysis methods could also be adapted to characterization of the root system from seedlings.

## Introduction

Roots provide essential functions including the uptake of water and nutrients (Gregory et al., 2009; Hammond et al., 2009; Lynch and Brown, 2012) for plant growth. They serve as storage organs (C and N principally), anchor the plants to the soil, produce hormones involved in development and response to stress and are the site of interactions with pathogenic, and beneficial organisms in the rhizosphere (Richards, 1983; Gregory, 2006; De Herralde et al., 2010). Root system is a complex three-dimensional (3D) structure exhibiting a specific spatial and temporal configuration of root types. The spatial distribution of all root parts, in a particular growth environment, is collectively referred to as Root System Architecture (RSA). RSA is dynamic and affected by the external environment. Roots indeed sense and respond to abiotic and biotic stresses (Malamy, 2005), and are able to communicate with the aboveground plants parts *via* signaling pathways, for example *via* hormones. In this context, studying the root system is fundamental to understand the global behavior of the plant (Smit et al., 2000). Moreover, studying the plasticity of root growth and development in response to abiotic and biotic factors provides opportunities for exploring natural adaptation and identifying beneficial root traits to enhance plant productivity in agricultural systems (Lynch, 1995; Kano et al., 2011; Grossman and Rice, 2012).

As the root system is complex to study in natural environment, it is necessary to have adapted devices under controlled conditions. Roots are hidden in most growth matrices, so destructive methods are generally used to evaluate root biomass at the end of experiments (Mugnai et al., 2008). Specific devices are required to investigate the distribution and dynamics of roots, as well as to evaluate their functioning. Containerized assay methods have facilitated such approaches in a smaller and reproducible manner. They include agar plates, hydroponics, paper roll methods, thin soil filled chambers (rhizotrons), soil filled tubes, large soil boxes, and field screens (Chen et al., 2015; Paez-Garcia et al., 2015, for review). Moreover, 3D imaging of the root system can be done by using X-ray computed tomography, magnetic resonance imaging or neutron tomography (Leitner et al., 2014; Metzner et al., 2015; as example). Hydroponics is one of the methods allowing growing plants without soil, hence potentially facilitating root observation (Conn et al., 2013; Mathieu et al., 2015). This method is easy to use and generally low cost, which gives it significant advantages. Rhizotron systems artificially restrict root growth to two dimensions only. They are subterranean rooms, laboratories, or plane containers with clear glass or plastic window to expose the soil for root visualization. Although they are very expensive to build and maintain, they provide a way of studying root systems throughout time, in a non-destructive way. Rhizotrons are widely used in root research as they provide an easy way to observe the growth and development of a large number of plants in a soil-like substrate (Chen et al., 2015) and allow a fine analysis of soil-root relationships. However, they do not allow the 3D growth and visualization of RSA. Over the last decade, various non-invasive imaging methods with higher spatial resolution have been developed to study 3D root development in soil with infiltration processes: magnetic resonance imaging (MRI) (Segal et al., 2008; Van As and van Duynhoven, 2013), X-ray computed tomography (CT) (Mooney et al., 2012; Metzner et al., 2015), or neutron tomography (Matsushima et al., 2008; Moradi et al., 2011).

Image-based methods (e.g., relying on the use of scanners or cameras) are mostly used for measuring the size, architecture, and other structural shoot and root traits. They allow hundreds of plants to be daily phenotyped, given the short time required for image acquisition (Clark et al., 2013; Adu et al., 2014; Le Marié et al., 2014; Slovak et al., 2014). Several software packages have been developed for root imaging and extracting quantitative data from captured images. ImageJ is an open source Java-based image analysis program, which is customizable with a variety of macros and plugins available, some of them written specifically for plant phenotyping applications. This program has been used, for example, in the IJ-Rhizo (Pierret et al., 2013) and SmartRoot software (Lobet et al., 2011). The Plant Image Analysis web site^1^ (Lobet et al., 2013) provides an on-line database and imaging solutions, commercial as well as open sources, for analyzing biology of plants. It provides a complete overview of existing software for root image analysis. Some of these tools require manual inputs from the user such as selecting points or tracing lines on the root, while others are automatic or semi-automatic. The most popular methods in root image processing are summarized in Table 1, according to the root characteristics requested in our study.

**TABLE 1:** Summary of currently available tools and the proposed method, for analysis of root images and the respective traits provided.

Software or Solution	Automation	Identification and separation of adventitious root	Adventitious root reconstruction	Trait provided ^(7)^						References
				ARL	DIA	LRN	LLRL	TNN	PNN	
ARIA	Automatic	+^(1)^	-	+	+^(2)^	-	-	-	-	Pace et al. (2014)
DART	Manuel	+	-	+	+	+	-	-	-	Le Bot et al. (2010)
EZ-Rhizo	Semi-auto	+	-	+	-	+	-	-	-	Armengaud et al. (2009)
RootNav	Semi-auto	+	-	+	-	+	+	-	-	Pound et al. (2013)
RootReader2D	Semi-auto	+	-	+	-	+	-		-	Clark et al. (2013)
Root System Analyzer	Automatic	+^(3)^	-	+	+	+	-	-	-	Leitner et al. (2014)
SmartRoot	Semi-auto	+	-	+^(4)^	+	+	-		-	Lobet et al. (2011)
RootGraph	Automatic	+	+^(5)^	+	+	+^(2)^	-		-	Cai et al. (2015)
RNQS	Automatic	+^(6)^	-	+	-	+	+		+	Remmler et al. (2014)
WinRHIZO	Automatic	+	-	+	+^(5)^	-	-		-	Arsenault et al. (1995)
Ascending Path	Automatic	+	+	+	+	+	+	+^(8)^	+^(8)^	Jeudy et al. (2016)

*For each available software, the table indicates the automation level, the possibility to identify and separate the adventitious root, to reconstruct the adventitious root and the trait provided.*

*^(1)^Dijkstra’s algorithm.*

*^(2)^Not detailed.*

*^(3)^Adventitious roots need manual initialization.*

*^(4)^Requires manual labeling of root types.*

*^(5)^Average value.*

*^(6)^Destructive method: requires to get out roots from the substrate.*

*^(7)^ARL: Adventitious Root Length; DIA: Diameter of the adventitious root; LRN: Lateral Root Number; LLRL: Longest Lateral Root Length; TNN: Total Number of Nodules; PNN: Primary Nodule Number.*

*^(8)^These traits have no biological significance for grapevine since it does not make such symbiosis.*

Identification of roots (adventitious and/or lateral) as distinct objects is an important goal for quantifying plant responses to various abiotic stresses including water stress and nutrient deficiency. For example, changes in nitrate and phosphate availability were found to have contrasting effects on primary root length and lateral root density, but similar effects on lateral root length (Linkohr et al., 2002). Most of the existing softwares can separate the primary roots from lateral ones, but with semi-automatic or manual methods (Lobet et al., 2013). Thus, they cannot be used efficiently for high-throughput usage in a root phenotyping pipeline. On the other hand, solutions such as ARIA, EZ-Rhizo, and RNQS were designed to analyze the roots of seedlings displayed in 2D scans (Pace et al., 2014; Remmler et al., 2014). The RNQS method requires to take out the plants from their pots and to clean them manually, which can result in slight plant destruction and thus in loss of data. Moreover, for adult plants, the root system can be anarchic and very complex, so it requires a more robust and refined method of analysis, and especially for the identification of adventitious roots. The different growing plant methods available for root imaging have advantages and limits. Their choice will depend on several factors, including the specific root traits of interest, degree of precision, desired timescale for sampling, infrastructure capacity, and costs.

The present study was focused on grapevine, (*Vitis vinifera* L.), a crop with high economic value facing major problems, and especially water stress associated with climate change. Viticulture also requires the development of more ecofriendly production systems. Identifying solutions to these problems and addressing this issue require experimentation in controlled conditions, integrating the root system. This is also needed for an increasing number of studies focused on the impact of biotic and abiotic stresses on vine development and physiology. Grapevine is propagated by cuttings (Waite et al., 2015). Roots arising from cuttings are called adventitious roots from which additional lateral roots are branching off. Such cutting process, associated with the possibility of using different genotypes of rootstock, impacts the development of its root system (Swanepoel and Southey, 1989). In this context, we have evaluated and compared three distinct methods allowing the study of architectural root traits of grapevine cuttings. We have evaluated two 2D- (hydroponics and rhizotron) and one 3D- (neutron tomography) imaging techniques for visualization and quantification of roots. We have also developed a robust and novel Matlab script for the automated high-throughput and high-resolution analysis of roots growing in rhizotron. Although this study was focused on grapevine the results obtained will be of interest for scientists working on other plants, especially those obtained from cuttings.

## Materials and Methods

### Grapevine Culture

For all cultivation methods, *V. vinifera* L. cv. Marselan (Cabernet sauvignon × Grenache) cuttings were obtained from herbaceous mother parents, as previously published (Trouvelot et al., 2008). Without further specification, herbaceous cuttings were always maintained in greenhouses at 23°C/15°C (day/night), under a 16-hour light photoperiod during all the experiment. To assess whether the cultivation system was suitable for grapevine rooting, the mortality rate was measured. It was based on the number of cuttings displaying necrosis signs developing on cutting ends and aborted growth of root and aerial parts, after 4 weeks of cultivation.

#### In Hydroponics Tube for 2D Imaging

**FIGURE 1:**
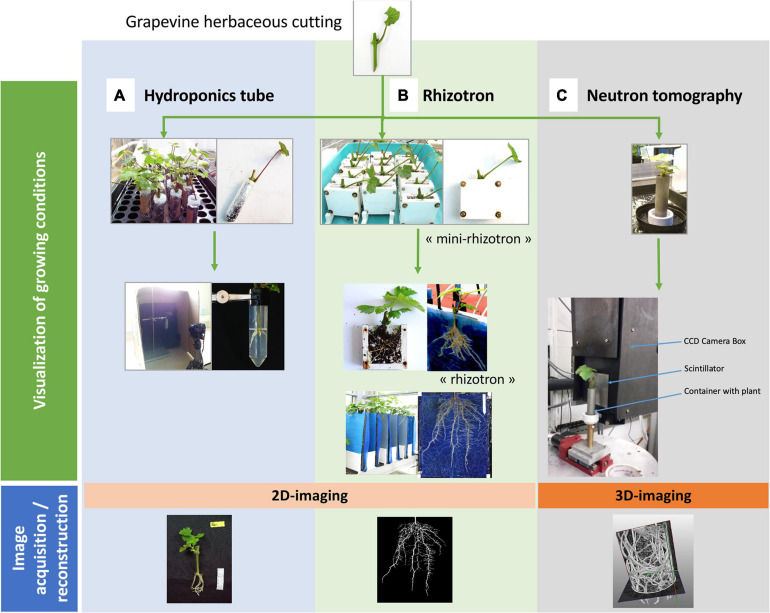
Overview of the different devices, growing conditions and image acquisition / analysis process. Grapevine herbaceous cuttings (cv. Marselan) were placed in three devices (top line: “Visualization of growing conditions”): **(A)** hydroponics tubes, **(B)** rhizotrons, and **(C)** aluminum cylinder for neutron tomography. They were then grown in greenhouse conditions and, depending on the method, some RSA parameters could be phenotyped. Quantitative parameters (i.e., projected root area) could be measured by image reconstruction and analysis (bottom line: “image acquisition/reconstruction”).

Hydroponics tubes consist on cap-free 50 mL Falcon^®^ tubes. Each tube received 50 mL of tap water, was wrapped in aluminum foil and was then closed on top by a piece of holed Parafilm^®^, through which an unrooted cutting (cut 7 cm down from the bud) was placed. A maximum of thirty tubes were held vertically on an empty seed starting tray and then placed in a polypropylene mini-greenhouse (56 × 36 × 25 cm, Botanic, France), with water at the bottom to keep a saturated humidity environment. This growing system, as the others, is illustrated in Figure 1A and its characteristics are presented in Table 2. Four weeks after the beginning of the experiments, cuttings were removed from the tubes and several parameters were determined: shoot height, fresh and dry weights of shoots and roots.

**TABLE 2:** Comparison of the three devices assessed: hydroponic tubes, 2D-rhizotron, and neutron tomography.

Characteristics		Hydroponic tubes	Rhizotron	Neutron tomography
Physical	Dimensions	3.3 × 11.5 cm	44 × 51.5 × 3 cm	3.5 × 10 cm
	Weight of one unit in use	65 g	6 kg	150 g
	Substratum (volume)	Liquid, solution (50 mL)	Membrane + Peat/ perlite (3/2, 2.8 L)	1 mm sand (50 mL)
Biological	Starting biological material	Unrooted herbaceous cutting (without unfolded leaf)	4 weeks pre-rooted herbaceous cutting (with 3 unfolded leaves)	3 weeks pre-rooted herbaceous cutting (with 2 unfolded leaves)
	Contact between roots and substrate	Yes	No	Yes
	Mortality during the experiment	5%	20%	Not evaluated
	Volume of irrigation	50 mL not renewed	300 mL per day	50 mL per day
Technical (for imaging)	Technique	Visible light	Visible light	Neutronic
	Sensor	Digital camera SONY- DSC-HX60	Scanner EPSON GT-15000	Camera CCD
	Resolution/ Level of precision	10 Mpixels (3648 x 2736)	300 dpi	6.8 mm/theoric pixel; 15.2 mm/pixel in the essay
	Type of data	2D imaging (pixel-based gray value images)	2D imaging (pixel-based gray value images)	180 2D images with a rotation of 1°
	Number of acquisition per plant	1 picture	2 scans	1 scan/ 1 3D reconstruction
	Field of view	Non-relevant	297 x 420 mm	8 x 80 mm
Phenotypic traits that could be determined	ARN	X	X	X
	ARL		X	X
	DIA		X	X
	LRN		X	X
	LLRL		X	X
	RSV			X
Logistic	Time required before mounting the test	10 min (cut of the mother plant)	4 weeks (time of pre-rooting)	3 weeks (time of pre-rooting)
	Time of assembly / disassembly of a unit	5–1 min	30 min–1 h	5–1 min
	Number of units available or manageable	60 manageable	12 available	As needed
	Occupied area for 10 units	0.02 m^2^	1.5 m^2^	0.05 m^2^
	Average acquisition time for a unit	10 s	15 min	4 h 40
	Maximum duration of an essay	4 weeks	5–6 weeks	Not evaluated
	Estimated average cost per unit per test	Less than 1 euro per tube	600 euros per unit	Zero*
	Specific remarks	–	–	3000 euros/day**

For each of the 3 devices, the table summarizes the main physical aspects, biological characteristics, technical parameters for image acquisition, logistic, and economic characteristics taken into consideration in this study.

*if official proposal selected by the large-scale infrastructure selection committee.

**if results are subject to confidentiality.

*ARN: Adventitious Roots Number; ARL: Adventitious Root Length; DIA: Diameter of the Adventitious Root; LRN: Lateral Root Number; LLRL: Longest Lateral Root Length;*

*TNN: Total Number of Nodules; PNN: Primary Nodule Number; RSV: Root System Volume.*

#### In Rhizotron for 2D Imaging

The rhizotron system was based on the Rhizotube^®^ design (Jeudy et al., 2016) except the fact that it was not cylindrical. Its characteristics are presented in Table 2. As it is a plane structure, placing directly an unrooted cutting in the device causes serious rhizogenesis difficulties. Moreover, placing cuttings that were pre-rooted in a pot causes their partial destruction as they developed in different directions. Rhizogenesis therefore needs to be carried out beforehand in a plane container to constrain newly formed roots to pre-adopt a 2D architecture. In this way, trials in rhizotrons were conducted in a two steps procedure (Figure 1B). In a first step, intermediary manufactured “mini-rhizotrons” (outer dimensions 8 × 8 × 3 cm; inner dimensions 6 × 7 × 1 cm) with substrate (peat/perlite, 3/2, v/v) were used for rhizogenesis. Grapevine cuttings (4–5 cm long down from the bud) were placed in “mini-rhizotrons” and grown during 4 weeks in a mini-greenhouse with saturated humidity, and watered daily to keep the substrate moist. At the end of this period, the grapevine cuttings were directly transferred in the final rhizotrons (step 2) as follows. The rhizotron is a methyl polymethacrylate structure (44 × 51.5 × 3 cm, laser cut by Lasertec, Arcelot, France) with two compartments separated with a blue nylon membrane (Jeudy et al., 2016): one for substrate filling (black plexiglas for the backside), and one dedicated for root growth (transparent plate for the frontside). In the first compartment, 2.8 L of substrate (peat/perlite, 3/2) were first spread and homogeneously pressed at the surface, in order to avoid irregularities in compaction. After the nylon membrane was placed, the 4-weeks old rooted cuttings were removed from the “mini-rhizotron,” roots were carefully washed with water before being positioned directly on the membrane (one cutting per rhizotron). Then the transparent plate was screwed on the device. An opacifier (dark polyvinyl chloride cover) was clipped on the outer face in order to avoid light exposure of the root system, and lastly, a small plastic bag was placed on the aerial part of the cutting in order to maintain humidity during the 1 week of recovery. Plants were automatically irrigated with two dripper tubes placed on both sides of the cutting, delivering in total 100 mL per irrigation time, three times a day, with nutritive solution (N/P/K 10-10-10, PlantIn, France) twice a week, and with same amount of tap water the rest of the week. Five weeks after the beginning of the experiments, cuttings were removed from the rhizotron and several parameters were determined: height of the shoot, fresh and dry weights of shoots and roots.

#### In Aluminum Cylinder for 3D Imaging

Since experiments performed with a natural soil present a too small contrast between roots and soil (Oswald et al., 2008; Moradi et al., 2009), fine sand was used as substrate for 3D neutron imaging (Figure 1C). Aluminum tubes (height of 80 mm and diameter of 22 mm, so volume around 30 ml) were then filled with aquarium sand (grain size 1 mm, Botanic SDS – IBP Archamps, France). As sand does not well keep humidity, it was difficult for the cuttings to take root in that context. Therefore cuttings of 4–5 cm long were pre-rooted in plugs of peat (2 × 4 cm Fertiss, Fertil SAS Boulogne Billancourt, France), for 3 weeks under saturated humidity, before being transferred in the aluminum cylinder with sand, and watered daily to keep the substrate moist. The tube is mounted on a 3D printed base that keeps it stable during culture and acquisition periods (Figure 1C). The characteristics of this system are presented in Table 2.

### Imaging of Grapevine Roots

#### 2D Imaging for Rhizotron and Adventitious Root Detection

The first image acquisition was run 4 days after transferring the cutting from the mini-rhizotron to the final rhizotron – a time period needed to guarantee correct plant recovery after transfer. This latter was checked by the presence of at least 1 cm long newly formed root and the presence of condensation in the plastic bag surrounding the cutting (witnessing foliar evapotranspiration). Image acquisition was realized by scanning the rhizotron directly through the transparent Plexiglas, using a scanner at 300 dpi (EPSON GT-15000, Seiko Epson Corp., Japan). As dimensions of the device were larger than the maximum area available on the scanner, the upper part and the lower part of the device were scanned in two halves. A common band area between the two images helped us to merge them numerically using a recomposition algorithm specifically developed to obtain a single image from two different ones. This algorithm is based in SIFT algorithm (Scale Invariant Feature Transform) (Lowe, 2004) which allows to extract key points using the RGB (Red, Green, and Blue) information. Ransac (Random Sample Consensus) (Fischler and Bolles, 1981) was also used on calculating homographies between both images. For the unfavorable cases (unperfectly flat), the discrepancy was around one pixel. Acquisitions were made every week for 5 weeks on 4 plants per replication, and experiments were repeated 3 times.

**FIGURE 2:**
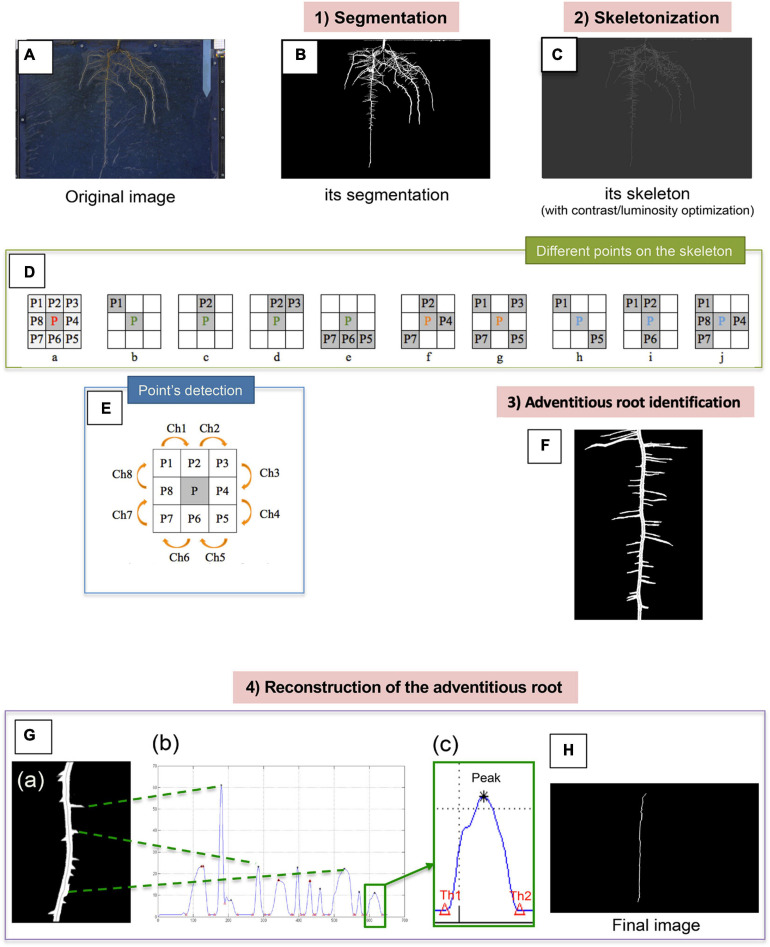
Summary of the different steps allowing the detection of the adventitious roots from the images acquired from the rhizotrons. The original image **(A)** firstly undergoes segmentation, which allows us to obtain a binary image **(B)**. Root skeletonization **(C)**. For each pixel in a digital binary image, 8 neighborhoods of a pixel P are defined: 4 points in direct direction and 4 other ones in diagonal direction **(D**a**)**. Different points on the skeleton can be distinguished: the endpoints (b–e), the junction points (f and g) and the normal points (h–j). Points detection **(E)**. Identification of the adventitious root **(F)**. Reconstruction of the adventitious root **(G)**. Example of image of the “pruned” root (a). Histogram of the vertical radius of the right part of the root (b) thresholds (Th1 = lower threshold, Th2 = upper threshold) around a peak (c). Final image of the adventitious root without lateral information **(H)**.

Merged images (Figure 2A) were then processed newly in a 4 steps image processing (Figure 2) resulting in: (1) segmentation, (2) skeletonization, (3) adventitious root identification, and (4) reconstruction of the adventitious root. The goal of image segmentation is to transform the images in order to facilitate their analysis to obtain more information. Here, we only used the Otsu’s method (Otsu, 1979), which is efficient in our study and fully automatic. In computer vision and image processing, this method is used to automatically perform clustering-based image thresholding or the reduction of a gray level image to a binary image. The algorithm assumes that the image contains two classes of pixels following bi-modal histogram (foreground pixels and background pixels). Otsu’s method is roughly a one-dimensional, discrete analog of Fisher’s Discriminant Analysis. Thus, this method of segmentation by thresholding allowed us to obtain a binary image with two classes: a white part which represents “Roots” and the black part which represents the background (Figure 2B).

The method of skeletonization by homotopic thinning (Iwanowski and Soille, 2007) has then been used. This method consists to look for the iterative erosion of the boundary of the object until obtaining a thin figure. At each step, the voxels whose deletion does not modify the topology of the object are cleared. The root skeleton obtained using this method is presented in Figure 2C. For each pixel in a digital binary image, the 8 neighborhoods of a pixel P are defined as follows: 4 points in direct direction (P2, P4, P6, and P8) and 4 in diagonal direction (P1, P3, P5, and P7; Figure 2Da). To search for the skeleton representing each adventitious root, lateral root or nodule, it is necessary to determine the type of each point on a morphological skeleton. In such a skeleton, three classes of points can be distinguished: the endpoints (Figures 2Db–e), the junction points (Figures 2Df,g), and the normal points (Figures 2Dh–j). In standard image processing, an endpoint in the skeleton of a binary image is an active pixel (gray pixel) that has only one active pixel in its neighborhood as shown in Figures 2Db,c) and 6 other possibilities in 45° rotation each time (Zhang and Suen, 1984). In addition, due to the natural root characteristics and the complexity of the skeleton image, the points of the Figures 2Dd,e were also defined as endpoints in our study (Lü and Wang, 1986). Normal points must have at least 2 pixels in their 8 neighborhoods, and up to 3 or 4 pixels. Once the different points categories were defined, a specific and new method has been developed to determine the class of a point which checks the successive changes of the active and inactive pixels to the 8-neighborhood (in binary image, it is the change between 0 and 1; Figure 2E). It consists in checking the successive change of the active and inactive pixels in 8-neighborhood (for a binary image, it is the change between 0 and 1). We check this change from P1 to P8 clockwise (Ch1∼Ch7), as well as between P8 and P1(Ch8). For example: if P1 = active goes to P2 = inactive or P1 = inactive goes to P2 = active, we note the change in pixels Ch = 1 (Figure 2E).

Once all the points have been identified and classified, a new method of identifying the adventitious root is proposed, called by the authors “Ascending Path.” The program starts from the bottom of the root, traveling in the upstream direction of the whole adventitious root, and finishes at the beginning of the root, regarding local image processing. This innovative method makes it possible to determine automatically the skeleton of the adventitious root (Figure 2F) and consists on the following steps:

(1) The starting point is determined with the lowest point and the breakpoint with the highest point in the skeleton image of the root.
(2) The program starts with the starting point, applying the ascending method, always taking the direction that goes up when it arrives at a crossing while traveling the skeleton. The indicator of this crossing is a junction point with at least 3 branches of different directions.
(3) To define the starting point and the breakpoint, as well as the recognition of the junction point, it is necessary to use the method of detecting the type of point presented above and tied to the Figure 2E.

The adventitious root can then be reconstructed from the identified skeleton in order to eliminate the lateral roots that are located on it. From the obtained skeleton of the adventitious root and, for each pixel, by examining each side (left and right) of the root mask bisected by the skeleton, our method reaches the boundary of the two classes (black and white) in a binary image, that is to say the change between 0 and 1, and with an accuracy of 1 pixel. An example of image of the adventitious root without lateral ones (i.e., deprived of them) is presented in Figure 2Ga. At each side of the adventitious root we can see the beginning of the lateral roots and of the potential nodules, and inside the adventitious root, the skeleton. The adventitious root was next cut in two parts from the skeleton in order to obtain a kind of histogram (for the right side Figure 2Gb). Each peak corresponds to the presence of a lateral root or a nodule, their classification depending on three parameters: height of peak, width of the peak, and area. Then, a low-pass filter was applied between the two thresholds (Figure 2Gc), considering the amplitude of each peak, repeating this step when there was no peak. Finally, an image was obtained which almost represents the adventitious root without lateral information (Figure 2H), with the same diameter as the original adventitious root. Images were acquired weekly, on 4 rhizotrons per replication, and experiments were repeated three times.

#### 2D Imaging for Hydroponic Tubes

Image acquisition started 1 week after the system was set up. Aluminum foils were removed and tubes were placed on a holder, in front of a black background. Images were acquired under natural light, by a camera (Sony DSC-HX60), placed on a tripod. Acquisitions were made every week for 4 weeks on 10 plants per replication, and experiments were repeated three times. Images were analyzed with the same method used for the rhizotron, described in the previous paragraph.

#### 3D Imaging by Neutron Tomography

The experiments were performed on the neutron imaging station, IMAGINE (Ott et al., 2015) at the French national neutron facility, the *Léon Brillouin Laboratory*, located at Saclay, France. The neutron-generating source is the ORPHEE reactor. The detector is constituted of a 50 μm gadolinium scintillator (RC Tritec, 2014) coupled to a Neo sCMOS (ANDOR) camera equipped with a sCMOS sensor (size of 16.6 mm × 14.0 mm, 2560 × 2160 pixels, so 5.5 Mpx). The size of one pixel is 6.5 μm × 6.5 μm. To reduce the noise, the camera was cooled down to −30°C. The camera was equipped with a 35 mm objective (Canon EF 35 mm f/2.0 IS USM). The sample holder was positioned at 5 cm away from the scintillator on a rotating table which allowed collecting 180 images in increments of 1°. Exposure time used for image acquisition was 80 s and ten images (with this exposure time) of open beam were collected for data normalization.

Every collected image was initially analyzed by Image J 1.48 (Abràmoff et al., 2004). We firstly cropped the image to select the object of interest (sample holder with the roots) and reduced the size of the data to analyze. We also used this software in order to remove noise (despeckle command) and white points due to gamma rays (remove outliers command, radius of 2 px; threshold of 50), to normalize the images by the open beam, and to correct the eventual tilt of the object of interest. Exported images were saved in.tiff format.

Octopus 8.7 (Octopus Imaging, Inside Matters, Belgium) was used to reconstruct the 3D image. This software allowed a last filtering step to remove rings due to scattering phenomena and building sinograms. Then, the reconstruction was realized, recording the output images in 32 bit. At the end of this step, the intensity values were set up between 0 and 1. After reconstruction with Octopus, a stack of 1074 slides (from the top to the bottom of the sample holder) was cropped and re-scaled (50%) on Image J. The Avizo Fire 9.2 (FEI, Hillsboro, Oregon, United States) software was then used to visualize the 3D volume. The quantification of the pixels corresponding to the roots on entire stack of reconstructed slides has been performed with the toolbox particle analysis of the Image J 1.48 software.

## Results

### Grapevine Root Traits Observed in Hydroponics Tubes

**FIGURE 3:**
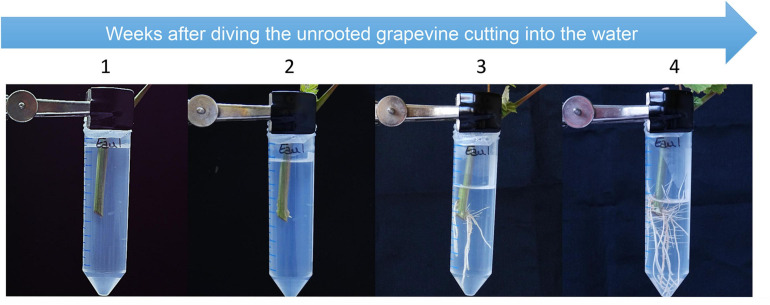
Grapevine herbaceous cutting growth and development in a hydroponic tube over time (i.e., weeks after diving an unrooted cutting into water). Cap-free Falcon^®^ tubes filled with water were closed on top by a piece of holed Parafilm^®^, through which an unrooted cutting was placed. Tubes were held vertically on an empty seed starting tray. The four pictures show the development of the root system for 4 weeks (1 image per week).

Unrooted grapevine herbaceous cuttings could develop easily in this culture system for 4 weeks. Indeed, the mortality observed during these experiments was low, evaluated between 3 and 5%. With hydroponics tubes, it was easy to visually follow the early root development of the cuttings over time (Figure 3), including the rhizogenesis. After 4 weeks of culture, roots occupied all the space available in the tube and the experiment had to be stopped. At this final time, the young plant could be recovered without injuring its root system, and different root traits such as the number of primary roots, average length of the adventitious roots could be observed. At this stage and in our experiment conditions, the root system weighed on average 388 ± 178 and 32 ± 16 mg in fresh and dry weight (±standard deviation from 3 biological repetitions), respectively.

The same algorithm as the one developed initially for the 2D-rhizotron system was then used to analyze the pictures acquired over time with the color camera for hydroponics tubes. Conversely, to 2D-rhizotron system, it was not possible to draw reliable data. Indeed, the fact that roots were immersed in a liquid and the container was cylindrical generated deformations in the images thereby acquired in 2D. To avoid the problem of cylindrical surface, focus could be done only on the central axis of the tubes. However, the main drawback concerned the transparency of the culture system which did not allow to catch the root in the center of the tube. Moreover, in some cases, the liquid could modify the optical way inducing a distorsion of the objects (i.e., roots).

### Grapevine Root Traits Analyzed in Rhizotron

**FIGURE 4:**
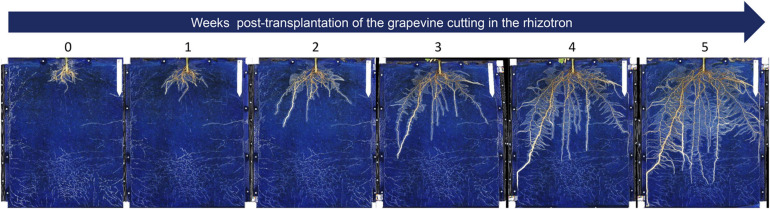
Root development over time of a pre-rooted grapevine herbaceous cutting grown in a rhizotron. The root development was followed during 5 weeks after transplantation of the cutting in the rhizotron (1 picture per week). The landmark at the top right side corresponds to 12 cm.

In this device, grapevine cuttings needed to be pre-rooted (during 4 weeks) in “mini-rhizotron.” As a consequence, at the time 0 of image acquisition, roots were already visible (Figure 4). In this device, one could easily follow the development of the root system during 5 weeks. At this stage, herbaceous cuttings were 9-weeks old. After this time, the roots gained the borders of the device and it was no longer possible to observe the root architecture reliably. At the final time (i.e., after 5 weeks of rhizotron culture), the root system weighed 15.4 ± 6.5 and 0.9 ± 0.5 g in fresh and dry weight (±standard deviation), respectively. The mean stem height was to 70.2 ± 20.6 cm and the shoot/root ratio was of 1.27 and 3.55 in fresh and dry weight, respectively.

**FIGURE 5:**
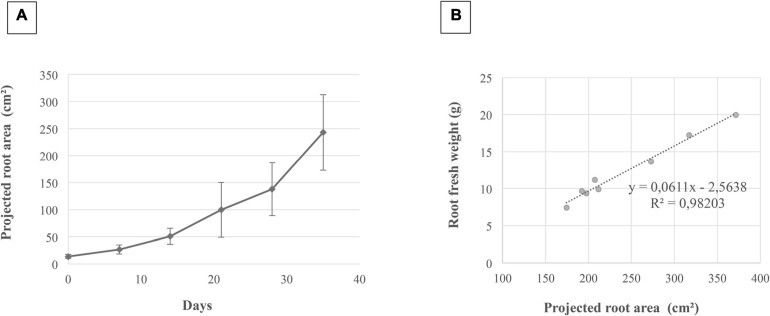
Correlation between the projected root area and root fresh weight. This correlation was determined for grapevine cuttings grown for 5 weeks in rhizotrons. Illustration of the projected root area calculated from root image analysis on 72 images **(A)**. Four images were analyzed for each time point, and three biological repetitions were carried out. Bars correspond to standard errors. Correlation between the projected root area and the root fresh weight **(B)**.

The images dynamically acquired were then analyzed by the procedure previously described (Figure 2) to determine the projected root area and the number of adventitious roots obtained. An illustration of the projected root area calculated during the time course and the correlation between this area and the root fresh weight is presented in Figures 5A,B, respectively. As shown in Figure 5A, the root system of the cuttings grown considerably in 5 weeks since its surface was multiplied by a factor 10. It was also observed that its growth dynamics was greater between 20 and 35 days than before. In addition, there was an excellent correlation (r^2^ = 0.98) between the projected root area (estimated by image analysis) and the evaluation of fresh root biomass (Figure 5B).

**FIGURE 6:**
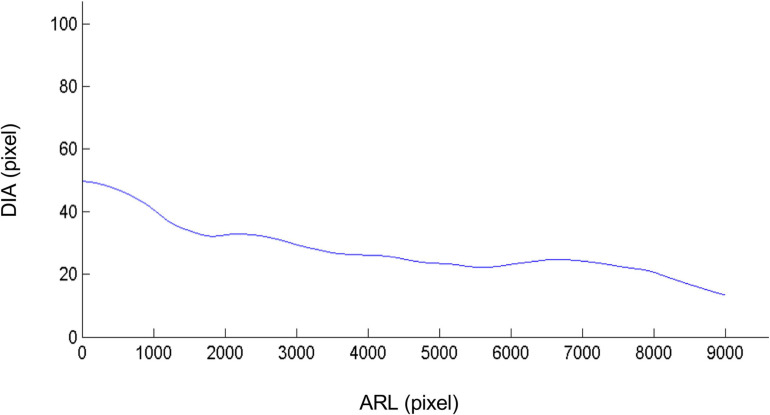
Local diameter in pixels of the adventitious root along its length. From the adventitious root identified and reconstituted by the algorithm, the calculation of its local diameter becomes possible by measuring the number of pixels occupied by the root throughout its length, allowing therefore the measurements of more diverse parameters such as the diameter of the root apexes in different stress conditions. In this case the root diameter is indeed decreasing as the distance from the cutting gets further. DIA: Diameter and ARL: Adventitious Root Length. The values of the diameter are after converted in mm using image calibration procedure: in our case 1 pixel is equivalent to 1 mm.

From the adventitious root identified and reconstituted, the calculation of its local diameter becomes possible and simple by counting the number of pixels occupied by the root throughout its length (Figure 6). It is therefore no longer an average diameter as with WinRhizo, SmartRoot or more recently RootGraph software. This local diameter makes it possible to calculate even more parameters such as the apical diameter of the roots and the unbranched apical zone of the primary root, etc., All the phenotyping traits, which are determined with the previous procedure, are presented in the Table 1, even if some of them present no biological significance for vine as they deal with nodules. Compared to semi-automatic or manual methods/algorithms (Table 1), the main advantages of our method concern the calculation time (4 min processing vs. 3 h for SmartRoot for example), the number of traits which can be determined and the automatic procedure.

### Grapevine Root Traits Observed by Neutron Tomography

**FIGURE 7:**
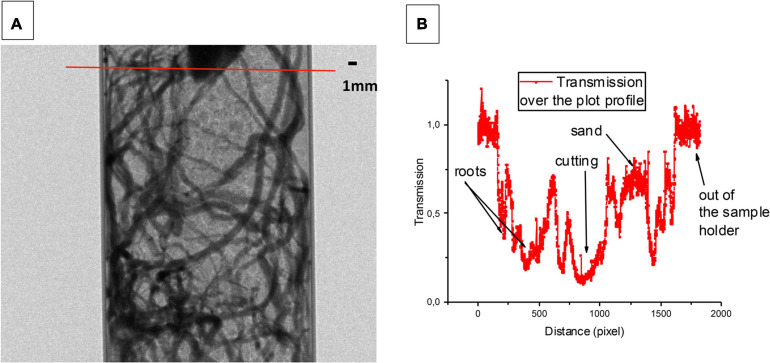
Neutron radiography of grapevine root and sand in an aluminum sample holder. The image **(A)** has been obtained with an exposure time of 80 s. The experimental resolution of the pixel is 15.2 μm which allows observing both the adventitious roots and its laterals. An example of a transmission profile is presented **(B)**. The transmission of the sand is around 0.8 whereas the one of the roots is going from 0.4 to 0.2. This difference of transmission between the sand and the roots corresponds to a contrast which allows to clearly distinguishing the roots from the soil (The image analysis has been performed by using the free access Image J 1.48 software).

We first measured, by neutron radiography, the attenuation of a neutron beam in a sample holder filled with sand and grapevine roots. Figure 7A showed an example of a recorded 2D image with the transmission values along a cross-section through the image (Figure 7B). There was a clear contrast between the sand and the roots. After the normalization, the image set had a mean transmission value of 1 for the open beam (out of the sample holder), while the sample area had different transmission values (ranging from 0.4 to 0.2) depending on their thickness and composition. The mean of transmission values observed for the sand was around 0.8, which highlight the visualization of the root system. The experimental spatial resolution of a pixel was 15.2 μm.

**FIGURE 8:**
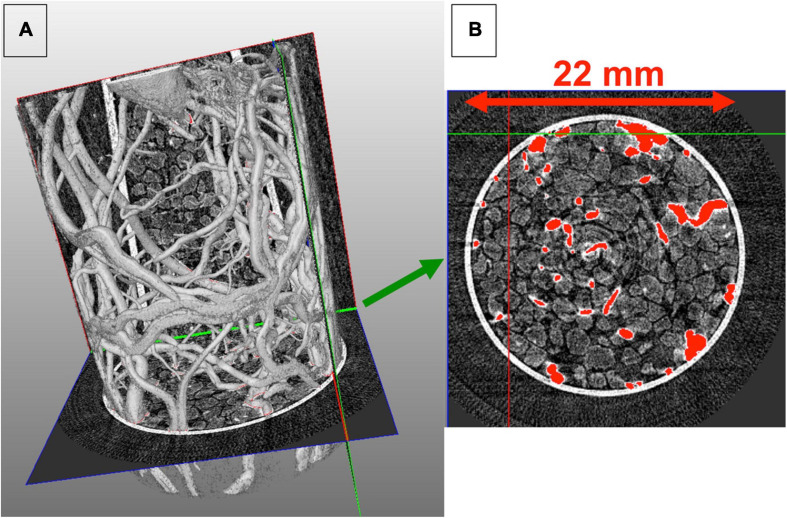
Reconstructed tomography obtained for grapevine roots. The grapevine herbaceous cutting has been grown in a sample holder filled with sand. The dense 3D architecture of the roots is clearly observed in **(A)**. A slide at the bottom of the sample holder is presented in **(B)**. A threshold has been used to specifically contrast the roots from the sand and the sample holder (this 3D volume visualization is performed with the Avizo Fire 9.2 software).

We recorded 180 images by rotating this sample by steps of 1°. As the exposure time for the acquisition of an image was 80 s and the time to transfer the data from the camera to the computer was 8 s, the duration of one tomography was around 4 h 30 s. After computational reconstruction, we could visualize the 3D organization of the roots and the sand inside the sample holder as illustrated in Figures 8A,B.

**FIGURE 9:**
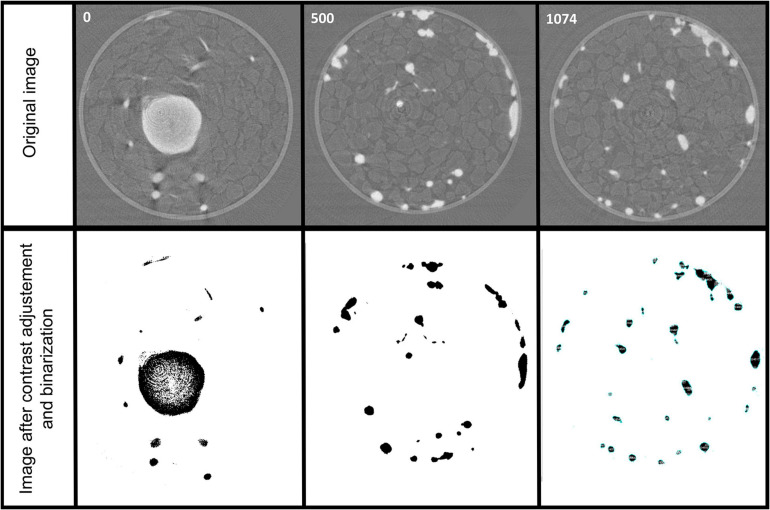
Image analysis for quantification of the root system. A contrast adjustment and binarization is performed on the whole stack of the 1074 slides obtained after reconstruction to specifically select the roots and quantify them by the particle analysis toolbox of Image J free software.

To quantify the root biomass inside the sample holder, we performed an image analysis using the IMAGE J free software from the stack of 1074 slides obtained after the reconstruction. After adjusting manually the contrast over the whole stack to specifically select the roots, a binarization of the images was applied also on the whole stack. Then the toolbox “particle analysis” of Image J was used to quantify the number of pixels corresponding to the roots. Figure 9 presents the results of these steps of image analysis for three different slides of the stack, one at the top (slide 0), one in the middle (slide 500), and one in the bottom (slide 1074). We found that over the reconstructed sample holder including the root system and the sand, 0.16% of the total volume corresponded to the root system (0.0486 cm^3^).

## Discussion

As there is a growing interest in phenotyping the root system of the vine under controlled conditions, it was necessary to identify the most suitable methodologies. Regarding the root phenotyping technologies available (De Herralde et al., 2010; Chen et al., 2015; Paez-Garcia et al., 2015), we decided to test and compare 3 methods: hydroponics and rhizotron, both with 2D-imaging and neutron tomography, with 3D-imaging, to highlight their main advantages and drawbacks. These methods are highly different regarding physical, biological, technological, logistic, and economic characteristics (Table 2).

### Hydroponics Tubes Allow to Easily Visualizing the Early Root Development of the Plant but It Is Not Possible to Quantify the Root Traits *in situ* Over Time

Hydroponics is frequently used for studies requiring control of nutrients and accessibility to the root system. Hydroponics is appropriate for cultivation of many plants and allows performing independent experiments in reproducible root-environment conditions. The advantages are its ease of use, cost and space-saving size. If necessary, the supply of water and nutrients can be adjusted easily. As unrooted herbaceous cuttings are used at the time 0 (i.e., cutting placed directly in water or in nutrient solution), this device is the only one among the tested ones that allowed us to follow the rhizogenesis process over time. However, the time course of experiments is limited to 4 weeks for grapevine cuttings in our conditions. Conversely to what is described in other studies (Chen et al., 2015; for review), quantification of RSA is not possible in a such systems due to root tangling in the liquid medium. In order to solve this problem, it may be relevant to evaluate the possibility of growing grapevine herbaceous cuttings in a device similar to rhizoponics described by Mathieu et al. (2015) for *Arabidopsis thaliana*. Indeed, such devices present the advantage to combine hydroponics and rhizotron. By this way, it allows non-destructive, 2D imaging of root architecture. This can be considered as an advantage since the root system is thus easily observed and harvested.

### 2D Imaging in Rhizotron Is Suitable for Grapevine Root Traits Quantification Such as Root Projected Area or Adventitious Root Length

Rhizotron allows the development of roots and shoots similar to those observed in pots and in “RhizoTubes” (Jeudy et al., 2016) for the same type of cuttings. Except WinRHIZO, all the softwares available nowadays are open source, but many of them are semi-automatic even manual like DART (Data Analysis of *Root* Tracings), SmartRoot, RootNav, and RootReader 2D. We thus proposed an automatic method for high-throughput and high-resolution root images characterization, using a specific pipeline.

Image segmentation is a crucial step in image processing, particularly in our Ascending Path method, and was used to identify adventitious roots of cuttings developed in rhizotron. It is an innovative result compared to previously published studies on grapevine with a similar device (Dumont et al., 2016). Several image segmentation methods have been reported in the literature. They can be divided in three main categories: segmentation by “Region” approaches, such as the Region-growing method (Adams and Bischof, 1994) or Split and Merge (Horowitz and Pavlidis, 1974); segmentation by “frontier” approach, such as the Canny filter method (Canny, 1986), and Segmentation by Thresholding, as Histograms (Jain, 1989). All these methods are different in terms of accuracy, complexity, and computation time. Threshold segmentation is a widely used technique for image segmentation (Gonzalez and Woods, 2002). This method uses the difference between the target area and the grayscale background, and then selects an appropriate value to determine the belonging class of each pixel in an image, in order to produce a corresponding binary image. The well-known and used method “AnalyzeSkeleton” (Fiji^2^; Author: Ignacio Arganda-Carreras) developed for detecting the three classes of points above, uses a simple counting of number of active pixels. It requires high calculation time and do not take into account all the root characteristics. In order to avoid these problems, and thus simplify the calculation time and identify the different points in a complex context, a specific method has been defined here. The development of this original method of image analysis makes this rhizotron system highly performing, and it merits adaptation to high throughput phenotyping (Jeudy et al., 2016) for grapevine root system observation. However, cutting manipulation and pre-rooting is time consuming.

Finally, following the development of the whole root system over the time could be of great interest to be sure to detect accurately the different phenotyping traits and to propose a universal method allowing these detections for different plants. This seems possible with the methods proposed in this paper but more experiments are needed to provide a global precise solution.

### 3D Imaging by Neutron Computer Tomography Is Relevant for the Quantification of the Root Volume Occupying the Container (Aluminum Cylinder)

Various methods allow the study of 3D root development. They permit observations of large objects with a field of view going from a millimeter to hundred centimeters. Moreover, they permit the visualization of opaque root structures. Series of projections are acquired and combined to reconstruct a 3D image of the root system. The imaging resolution is usually around few micrometers depending on the size of the observed object. In previous experiments, MRI has been used to study water infiltration toward root-colonized soils (Segal et al., 2008). However, full exploitation of MRI methods is handicapped by the high content of paramagnetic particles and the high heterogeneity of structure and geochemical composition. The last 30 years application of X-ray Computed Tomography (CT) has demonstrated considerable promise for root visualization studies. Micro CT scanners are now able to achieve high resolutions (50 μm), which enhanced capability to detect fine roots. However, the overlap in the attenuation density of root material and soil pore space (even more when full of water) is still a limitation to the study of water infiltration and root-soil interactions. Neutron radiography measures the attenuation of neutrons through a medium. Neutrons interact with atomic nuclei and this interaction does not show periodic regularity with the atomic number. They are particularly sensitive to light elements such as hydrogen and lithium, while being relatively insensitive to metals such as aluminum (Kang et al., 2013). Neutrons are therefore ideally suited to deeply penetrate most common materials but are strongly attenuated by those containing hydrogen such as water. Previous studies (Moradi et al., 2009, 2011) have used neutron radiography (NR) to study *in situ* root developments in soil of different textures. They demonstrated that sandy soil was the best substrate to obtain a good contrast for the root visualization. They also used neutron tomography (NT) to quantify and visualize the water content in the rhizosphere of chickpea, lupin, and maize, 12 days after planting. NT of the root-soil interface showed an increase in soil water content close to the roots. Both adventitious roots and lateral roots showed higher water contents in their rhizosphere compared with bulk soil.

Through this first study of the grapevine roots grown in sand in a sample holder of 22 mm of inner diameter and 80 mm long (large object), we have shown that it is possible to visualize grapevine root network by using neutron tomography. A 3D imaging of the root system could be obtained at a resolution of few micrometers (15.6 μm). We have been able, using a very simple and preliminary image analysis (with free software), to quantify the percentage of root occupation in the total volume of the cell holder. To go further on the root system analysis, one should perform a deeper image analysis with a segmentation of the root regarding their sizes (number of pixels) to classify them into adventitious or lateral roots. One could also extract the mean size of these different root classes by image analysis (Metzner et al., 2015). Also as perspective one can use neutron radiography technique to monitor water distribution and root growth simultaneously, making it suitable for studying root-water relationships in soils (Oswald et al., 2008; as example).

## Conclusion

This study highlights the advantages and limitations of three devices specifically developed and/or adapted to phenotyping of grapevine roots. Each of them has interest, depending on the objective of phenotyping and on the number of plants needed for it. Even if hydroponics does not allow precise root quantification, it consists on a rapid approach with a low cost and provides rapid first information. Despite being more complex, 2D-rhizotron is particularly suitable for grapevine root traits quantification such as root projected area or adventitious root length. Finally, 3D imaging by neutron tomography is the most complex device but is the most relevant for quantifying the root volume occupying the substrate. To our knowledge, it is the first time that this last device is tested to phenotype grapevine roots.

In the case of 2D-rhizotron phenotyping, we have developed a new image analysis Matlab script adapted for rhizotron for identifying adventitious roots as a feature of root architecture. Calculation time for each image takes between 3 and 5 min automatically, whereas for the standard software generally available it takes more than 30 min. Thus, this method is automatic, fast and unsupervised, and allows high-throughput adventitious root parameters determination with high resolution images (144 Mpixels). Moreover, none of the other software can provide all the information on the RSA, as mentioned in Table 1.

Even if image acquisition is not the same for the three devices tested and previously described, image processing could be similar using this new method proposed with “Ascending path” procedure and identification of the different points on the skeleton.

Although this study was focused on grapevine, it has also interest for other plants, especially perennial ones.

## Data Availability Statement

The original contributions presented in the study are included in the manuscript/supplementary material, further inquiries can be directed to the corresponding author/s.

## Author Contributions

MA, M-CH, ST, and YK designed the study and coordinated it. YK carried out hydroponics tubes trials, as well as the rhizotron ones in collaboration with EB. CS managed the phenotyping platform and helped us to adapt the Rhizotubes device in a plane manner. SH developed the procedure to detect adventitious roots. AL tested it on images acquired in the hydroponics tubes device and developed the recomposition algorithm. FC coordinated the image analysis work. YK, EB, MA, CL, and FO realized the neutron computed tomography experiments. CL analyzed the results. ST drafted the manuscript. CL, FC, MA, and YK helped to draft it. All the authors read and approved the final manuscript. All authors contributed to the article and approved the submitted version.

## Conflict of Interest

The authors declare that the research was conducted in the absence of any commercial or financial relationships that could be construed as a potential conflict of interest.

## Footnotes

1 http://plant-image-analysis.org

2 http://imagej.net/AnalyzeSkeleton
